# The impact of IoT security labelling on consumer product choice and willingness to pay

**DOI:** 10.1371/journal.pone.0227800

**Published:** 2020-01-24

**Authors:** Shane D. Johnson, John M. Blythe, Matthew Manning, Gabriel T. W. Wong

**Affiliations:** 1 Dawes Centre for Future Crime, University College London, London, England, United Kingdom; 2 ANU Centre for Social Research and Methods, The Australian National University, Canberra, Australia; King Saud University, SAUDI ARABIA

## Abstract

The Internet of Things (IoT) brings internet connectivity to everyday electronic devices (e.g. security cameras and smart TVs) to improve their functionality and efficiency. However, serious security and privacy concerns have been raised about the IoT which impact upon consumer trust and purchasing. Moreover, devices vary considerably in terms of the security they provide, and it is difficult for consumers to differentiate between more and less secure devices. One proposal to address this is for devices to carry a security label to help consumers navigate the market and know which devices to trust, and to encourage manufacturers to improve security. Using a discrete choice experiment, we estimate the potential impact of such labels on participant’s purchase decision making, along with device functionality and price. With the exception of a label that implied weak security, participants were significantly more likely to select a device that carried a label than one that did not. While they were generally willing to pay the most for premium functionality, for two of the labels tested, they were prepared to pay the same for security and functionality. Qualitative responses suggested that participants would use a label to inform purchasing decisions, and that the labels did not generate a false sense of security. Our findings suggest that the use of a security label represents a policy option that could influence behaviour and that should be seriously considered.

## Introduction

The Internet of Things (IoT) brings internet connectivity to everyday electronic devices, allowing them to collect and share data over networks. In doing so, it promises to improve their functionality, efficiency, and our interaction with them. IoT devices can range from speakers, to app-controlled burglar alarms to wearable health monitors to connected cars, and they have the potential to come together to form smart homes, smart offices and, ultimately, smart cities [1]. Currently, there is an estimated 6.4 billion connected things online which is expected to reach 20.8 billion by 2020 [2].

However, the IoT brings unique challenges with respect to cyber security. This is in part due to the fact that many are low powered devices with limited processing capacity [3] that are deployed in uncontrolled environments [4]. Moreover, securing the IoT is a non-trivial task, given the heterogeneity of devices [1]. However, it is also fair to say that the security of IoT devices has been a low priority for manufacturers, with the lack of even basic in-built security features (such as software updates and the use of unique passwords) being well-documented [5,6]. For example, research has shown that—by taking advantage of hardware or protocol flaws—attackers can exploit man-in-the-middle attacks to intercept communications from and to wearable devices to steal personal information, invading user’s privacy and exposing them to the risk of identity theft [7]. Attackers can also take advantage of the weak authentication and encryption protocols used in many cameras enabling them to eavesdrop on camera streams [8], again raising privacy concerns and creating opportunities for crime [9]. Such concerns may impede consumer confidence in, and the uptake of, the benefits the IoT promises to deliver [4]. Consequently, addressing these concerns is of paramount importance.

While there are many applications of the IoT, in this article, we focus on the consumer IoT. That is, devices that may be found around the home and purchased and owned by regular citizens. Security and privacy concerns have led to low consumer trust in the IoT with only 9% of consumers trusting IoT products, although 42% continue to use and recognise the value the IoT brings [10]. To build consumer trust in the IoT effectively requires the security and privacy concerns discussed to be addressed, and for consumers to perceive this to be the case [4]. In this paper, we focus on the use of labelling schemes as a mechanism for encouraging manufacturers to improve the security and privacy features of devices and to enhance consumer trust in the IoT.

Presently, there is little incentive for manufacturers to produce secure products and there are “information asymmetries” between producers and consumers. These asymmetries are due to a lack of accessible information about a product’s security which makes it difficult for consumers and retailers to assess the security of products. For example, recent research involved a review of the online materials associated with 270 IoT devices to see what consumers could find out about the security of devices prior to their purchase. The authors found that little information was available to consumers [11]. These asymmetries manifest as risky purchasing decisions that cannot be informed by a consideration of device security. Offenders can, of course, benefit from this due to the increase in crime opportunities associated with the proliferation of insecure internet connected devices, and the fact that details of insecure devices can be found on the dark web. One route to reducing these asymmetries and to enhance consumer trust is to develop a labelling scheme to inform consumers and retailers about the security afforded by a device, which can be displayed on the front of packaging, on a retailers’ website, or communicated through other means.

Policymakers have expressed an interest in the role of labelling schemes to provide consumers with the information necessary to make more informed choices about the security of a device [12]. Calls for labelling schemes have also been proposed by industry [13] and certification bodies [14]. However, the format of these labelling schemes differs across proposals and fall into three broad types: binary “seals of approval” where products are certified to a security standard; “informational” labels that would communicate important information around security/privacy across a series of dimensions; and, graded schemes that would measure the security of a device across a continuum–graded schemes are often colour, letter or star coded to further aid their interpretation. However, what is not understood is what type of labelling scheme may have the biggest impact on consumer behaviour in the context of IoT security. Another identified gap is that we lack an understanding of how consumers value security alongside other attributes of IoT products (such as their functionality) and how much they would be willing to pay for a secure product. We aim to address these research gaps in the current study by examining how labels that demonstrate a device’s security posture might affect choice and willingness to pay for a range of internet connected devices.

Labelling schemes are a popular policy choice for informing consumer decision making. However, their shaping of consumer behaviour at the point of sale is limited by a number of factors including time pressure, comprehension difficulties and competing priorities (such as functionality, price and promotions) [15]. As consumers have a limited cognitive budget to expend at the point of purchase, choosing the most optimal design of a labelling scheme is necessary to nudge consumers effectively. Research has evaluated the effectiveness of existing labelling schemes in food and energy sectors to evaluate the three types of label [16]. This suggests that informational labels are the least understood, particularly for individuals of lower socio-economic status. Seal of approval labels, although preferred by consumers for their simplicity, are associated with unintended consequences such as dichotomous thinking (e.g. wrongly assuming that a product with a label is better than one without) and halo effects (e.g. a false sense of security). Finally, graded schemes have been shown to have a greater impact on consumer behaviour because they invoke the *affect heuristic*, a mental shortcut people often use when making decisions. For example, the green to red colours, or A to F grades that are commonly used for such labels are scales that people are regularly exposed to, and their familiarity strongly influences people’s behaviour in the presence of lots of information. Although the effectiveness of a labelling schemes is dependent upon its regulatory underpinnings and whether its presence is mandatory, there is currently no published research that assesses the impact of a security label for internet connected products. The current study seeks to explore labelling formats within this context. Ultimately, an effective label is one that helps consumers distinguish between a secure and less secure product, instils feelings of trust, and does not cause comprehension difficulties for different demographics and is easy to understand.

Willingness to pay (WTP) is the maximum amount a consumer will pay for a product [17]. WTP is useful for understanding consumer demand and can be used to inform tactical pricing and the development of new products (and services). Presently, not all manufacturers ship IoT products with security built-in, and those that do often leave security concerns until the final stages of product development [18]. Although not explicitly tested, there is an assumption that consumers are not willing to pay for greater security in IoT products [19]. Assessing WTP would thus allow us to estimate the highest price a consumer would pay for a product with greater inbuilt security. Previous research has shown that consumers are willing to pay for more online security measures to protect their home computers [20,21]. However, in the IoT context, consumers’ mental models of risk and IoT devices may differ as these once everyday objects, such as thermostats and watches, were not conventionally susceptible to online risks. Moreover, research has shown that WTP judgements are context sensitive [22]. As such, consumers may be willing to pay more for certain classes of devices, such as those that are linked to physical security (such as security cameras) or to safety critical services (such as thermostats).

It is possible that cybersecurity concerns and behaviours vary with age. However, previous research paints a contradictory picture of the link between age and cybersecurity. Some studies suggest that young people are more vulnerable to cyber threats [23,24] and disclose more personal information online than others [25,26]. Other work suggests that while children engage more online, they have greater security concerns than older people [27,28] and balance their personal disclosure on social media with their privacy needs [29].

Older adults, on the other hand, have been found to be more privacy-aware [30] but less willing to adopt some cybersecurity practices such as the use of PINs or biometric authentication [31], and also engage in less privacy protective behaviours on social media sites [32]. In the context of the purchasing of secure computing products, there is no research on the effects of age but research on consumer buying behaviour suggests that, due to reduced cognitive ability, older adults may rely more on heuristics in their purchasing decision making [33]. Work on food labels has found that older adults struggle to interpret information accurately [34] but other studies have found no effect of age on labelling interpretation [35]. As such, it is unclear whether age can be expected to play a role in the degree to which a person responds to the presence of a label.

When consumers are asked about the IoT, security and privacy concerns are repeatedly referenced as a key barrier to adoption [10,36] yet many consumers will not disconnect due to the affordances the technology brings [10]. This is the classic “privacy paradox” in which people report having privacy concerns but often do little to protect their privacy [37]. With the IoT, this is in part due to these protective actions being unachievable for the majority of consumers [38] and there being no clear mechanism for them to protect themselves at the point of purchase (e.g. a labelling scheme that would assist in purchasing decisions [39]). Another issue is that intention only accounts for 1/3 of actual behaviour [40]. People who have greater concerns may be more susceptible to the influence of a labelling scheme than those who have lesser concerns. However, this paradox makes testing this relationship difficult [37]. Whilst concerns are important, behaviours are the actions that consumers engage in to protect themselves from cyber threats. A more effective alternative is to therefore use a behavioural measure [41] and to explore whether those who engage in more protective cybersecurity behaviours are influenced more by a labelling scheme than those who do not. Consequently, we seek to explore whether those who engage in greater cybersecurity behaviours are influenced more by an IoT security label.

The overall aim of the current study is to assess the effectiveness of three different labelling schemes (Graded, “Seal of approval” and informational) in nudging consumers towards “secure” products and away from products that offer no assurances around security. The extent to which WTP is influenced by current security behaviour, as well as the age and gender of participants, and their self-reported security behaviour is also investigated. The study thus addresses the following two main research questions:

1. *What (if any) labelling scheme has the biggest impact on consumers purchasing decisions*?
2. *How much are consumers WTP for the security of domestic IoT devices*?

To address the research questions, we use a discrete-choice experiment (DCE). DCEs are a systematic method for eliciting trade-offs to quantify the relative importance that consumers assign to various product attributes (such as functionality and price). Currently, we cannot estimate consumer’s actual preferences around IoT purchasing as these data do not exist. Furthermore, there is no current implementation of an IoT labelling scheme on the market and so we need to assess their utility in a hypothetical situation by exploring consumers stated purchasing preferences. In a DCE study, participants are provided with purchasing options for which product attributes vary and asked to choose their preferred option from two or more alternatives. In the current study, these attributes were the functionality of the product, product price and the presence or absence of an IoT security label. The premise is that with each choice people make, they will seek to maximise their utility (i.e. pick choices that yield the greatest satisfaction for them). Alternatives are described by varying levels of key product attributes which represent factors included in a “utility function”. For example, in a hypothetical DCE examining two levels of both functionality (standard or premium) and price (£100 or £130), a potential choice set presented might be a standard product priced at £100 versus a premium product priced at £130. Participant’s choices are assumed to be dependent on the levels of the attributes and the relative value that they place on each one. By presenting participants with multiple scenarios with differing levels of attributes, and asking them to choose one, DCEs are used to generate data that–through statistical analysis–are used to estimate which attributes drive consumer preferences and the trade-offs they make between attributes.

What consumers are willing to pay for a particular good or service can also be estimated using a DCE design when price is included as an attribute. In doing so, one can estimate the marginal WTP for the tested attributes, as well as a total WTP for all attribute levels [42]. A DCE design provides richer information than other methods for eliciting WTP as it accounts for how individual attributes affect utility [43]. In assessing WTP, we can estimate (for example) how much people are willing to pay for a product that provides security assurances via a labelling scheme, and do so without asking them about this directly.

In addition to the above research questions, a potential concern with the use of a labelling scheme is that consumers may develop a false sense of security, believing that a device that carries it is immune from hacking. Since no device can be immune from attack, a potential unintended consequence of such a label is that consumers will not take relevant precautions to protect themselves from online risks or will not trust the label if they discover that devices which carry it have been compromised. The third research question addressed here is thus:

3 *To what extent do consumers perceive that a device that carries a security label would be resistant to hacking*?

We examine this question, and gauge participant’s perceptions of the different labels, using a simple survey administered alongside the DCE. As such, in this paper, we make the following contributions:

- (Alongside functionality and price) we examine the effect of three different labelling schemes (informational, binary and graded) on consumer (hypothetical) purchasing decisions, testing variations of two of them.
- We test the hypothesis that those who already engage in more online security behaviours (estimated using a behavioural measure) will be more influenced by security labels.
- We test whether the effect of the labels interacts with age or gender.
- We estimate consumer WTP for products that provide assurances about security.
- We explore consumer perceptions of the labelling schemes, including their ease of interpretation, whether consumers would use them to guide purchasing decisions, and if they generate a false sense of security.

## Materials and methods

### Design

This study adopts a stated preference approach to understand consumer purchasing behaviour to estimate the influence of three attributes: labelling, functionality and price. This design was replicated across four types of devices (security camera, Smart TV, smart thermostat, and a wearable)—chosen to represent a mixture of high and low-cost devices that have differing levels of safety criticalness -, and seven labelling conditions (discussed below).

Table 1 provides a summary of the devices and their characteristics. The first two factors had two levels–presence or absence in the case of the label (for more detail, see below), and standard or premium in the case of functionality. Product functionality was based on a qualitative analysis of IoT devices sold on the websites of three UK retailers (Amazon, PC World and John Lewis). For each of the four devices, the most frequently mentioned functions were identified and those that commonly recurred were defined as “standard”, while those that differentiated products were classified as premium (see Table 1). For price, there were five levels: baseline, 10%, 20%, 30% and 40% more expensive. The baseline price was equal to the average of the sampled devices from the retailers described above.

**Table 1:** Study attributes in choice sets and levels.

Attribute	Level	Security Camera	Smart TV	Smart Thermostat	Wearable
**Functionality**	Standard	Recording Quality: 1080p HD, Night vision: Yes, Sound/movement detection: No	Display resolution: Full HD 1080p, Freeview HD: Yes, Streaming: No	Touch screen interface: No, Auto-schedule: Yes, Hot water tank control: No	Smartphone notifications: No, Health and fitness tracking with heart rate monitor and GPS: Yes, Battery life: 3 days
	Premium	Recording Quality: 4k, Night vision: Yes, Sound/movement detection: Yes	Display resolution: 4K Ultra HD, Freeview HD: Yes Streaming: Yes (Inc. Netflix, YouTube)	Touch screen interface: Yes, Auto-schedule: Yes, Hot water tank control: Yes	Smartphone notifications: Yes, Health and fitness tracking with heart rate monitor and GPS: Yes, Battery life: 7 days
**Label**	Present	-	-	-	-
	No label present	-	-	-	-
**Price**	1	£99.99	£350.99	£159.99	£69.99
	2	£109.99	£385.99	£175.99	£76.99
	3	£119.99	£420.99	£191.99	£83.99
	4	£129.99	£455.99	£207.99	£90.99
	5	£139.99	£490.00	£223.99	£97.99

#### Types of labelling

**Fig 1:**
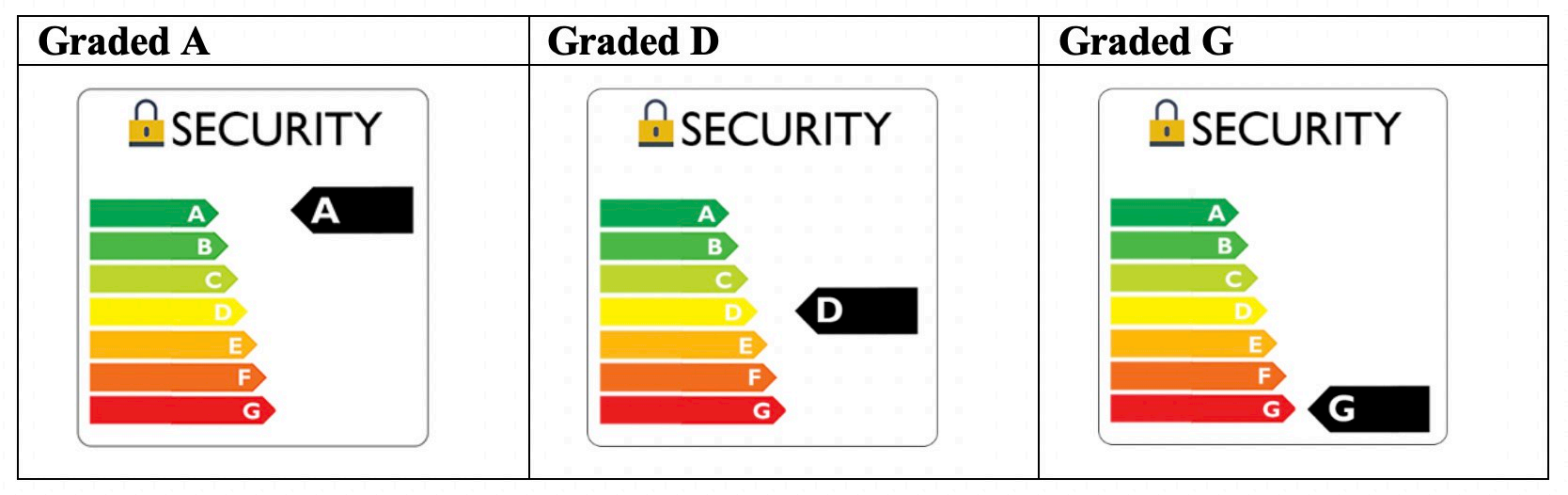
Graded label.

The labelling formats were chosen based on three general types that have been proposed by industry, government and academia, as follows:

**1) A graded label**: This was based on the labeling scheme used to convey information about energy consumption for electronic goods. This scheme rates the energy efficiency of a product from A to G, with A being most and G being the least efficient. These markers are paired with a colour cue to indicate performance, with greener products (e.g. A) indicative of greater performance than red (e.g. F). Here, we used an A-G grading and green to red colour coding—to indicate more or less “secure” devices—as these are known to influence consumer behaviour through the *affect heuristic* (for a recent review, see [16]. As shown in Fig 1, in the current study, we assessed three implementations of this label (Grade A, Grade D and Grade G).

**2) “Seal of approval” label**: To examine the effect of a binary “seal of approval” label, we chose to use an existing labelling scheme (see, https://www.securedbydesign.com) that has been used for physical security and has been shown to be effective in driving market changes in the physical security arena. As the label is binary, we needed to only assess one implementation of this label.

**Fig 2:**
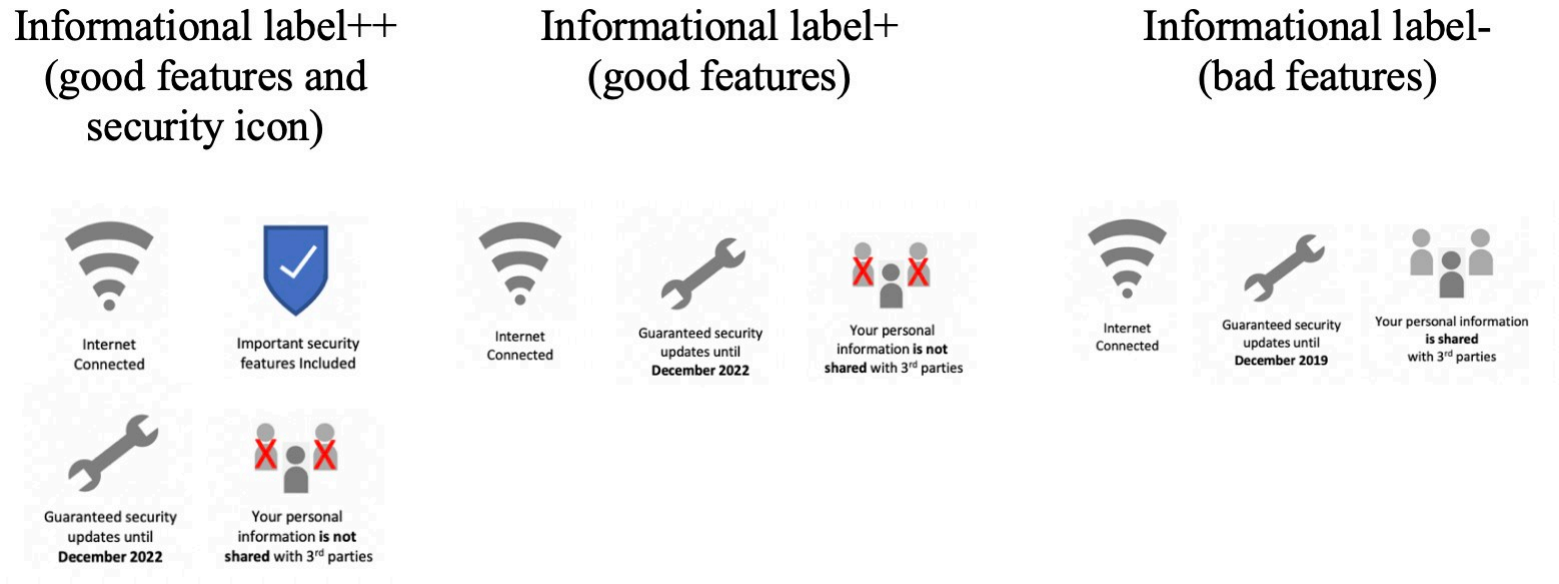
Informational labels (images are similar but not identical to those used in the study and are therefore for illustrative purposes only).

**3) Informational label**: We chose to emphasise three key dimensions of this type of label (see Fig 2). These were the device’s internet connectedness, security support period and whether information is shared with third parties. The first two dimensions were based on label content as outlined by the UK Government Department for Digital, Culture, Media and Sport [5]. The third dimension was rated as a priority by participants in a previous study and is a consistent privacy concern for consumers [44]. Given the interpretive nature of the dimensions, we chose to assess three implementations of this label. An “*information label for a device with good features*” which had a long support period (until December 2022) and did not share personal information with third parties. An “*information label for a device with bad features*” which had a shorter support period (until December 2019) and did share personal information with third parties. As these are proximate indicators of security, as a third implementation, we also explored an information label with a security icon–“*information label for a device with good features and security icon”*.

In total, then, there were seven labelling conditions, and the labelling condition was a between-subjects factor.

#### Choice sets

For every device type, we constructed choice sets for each participant. For each choice, which was intended to represent a purchasing decision, participants were asked to select from one of four options. Three of these described the features of a device (i.e. whether it had a label, the level of functionality and its price) while the fourth was an “opt out” that allowed participants to choose none of the options. For each option, there were 20 possible combinations of the three factors—2 (label or not) x 2 (standard or premium functionality) x 5 (price levels). As there were three options for each choice, this produces a total of (20x19x18/6 =) 1,140 possible (device) combinations for consumers to contrast, which is clearly too many. To make the number of combinations tested feasible, we sample from all those possible using the D-efficient design algorithm in STATA13SE, and generated 10 choice sets per device. Participants were asked to complete the choice sets for two devices and were always allocated to the same labelling condition.

#### Sample size estimation

The minimum sample size required to minimise the risk of Type II statistical error (not detecting an effect where one exists) was computed using the equation presented in [45]. According to these calculations, a minimum of 83 participants were required per condition. However, a review of the literature revealed that most DCEs employ sample sizes in the range of 100–300 [46] and, prior to testing it is difficult to estimate how many participants will select the “opt out” choice—which reduces statistical power. Rather than risk having an underpowered design, we aimed for a sample size of around 200 per condition. As a further precaution, we ran Monte Carlo simulations to generate synthetic data to confirm that coefficients could be correctly estimated using the D-efficient design and a range of sample sizes, including that selected.

#### Pilot study

Prior to conducting the main study, a pilot study was completed and the design adjusted in the following ways: 1) In the pilot study, participants were provided with two alternatives for each choice set (plus the “opt out” option). As discussed above, we increased this to three choices (plus the “opt out”) in the final study. This was to make the task more realistic and to make it less obvious that the study concerned the impact of the security label on consumer choice. That is, with three options, for some choice sets, participants would see two products with a label (and one without), but for others they would see only one with a label (and two without). This contrasts with the pilot study design for which participants were always asked to choose between one device with and one without a security label; 2) In the pilot study the four products tested included a baby monitor. However, because not all those who participated in the study would be parents, we decided that it would be sensible to exclude this device from the main study (and replace it with a Smart TV); and, 3) As stated preference studies ask participants about hypothetical scenarios, there is a risk that those who participate will overstate their willingness to pay for a particular good or service (Loomis, 2014). This risk is of particular concern for contingent valuation studies—for which participants are directly asked how much they would be willing to pay for a good or service—but we wanted to minimize this risk here too. To do so, we employed a cheap talk intervention, which is used to explicitly advise participants of the potential for this type of hypothetical bias with the aim of reducing it [47].

#### Security behaviour

To estimate participant’s existing security behaviour we used the 16-item Security Behaviour Intentions Scale (SeBIS) [48], which consists of four subscales (attitudes towards choosing passwords, device securement, staying up-to-date, and proactive awareness). Items were measured on a five-point scale of 1 (never) to 5 (always), and the full scale had a reliability of α = 0.79 and its sub-scales: attitudes towards choosing passwords α = 0.71, device securement α = 0.65, staying up-to-date α = 0.68, and proactive awareness α = 0.63.

#### Participants

In total, 3000 UK adults, recruited through the online panel prolific.ac, took part in the study. Eighty-two participants were removed for failing attention checks (see below). Participants were paid £1.00 to take part and to be eligible for participation, they had to be aged 18 or above and live in the UK. Table 2 provides descriptive statistics for the sample.

**Table 2:** Participant demographics.

Participant characteristic	
**Gender**	**Total (%)**
Male	1007 (35%)
Female	1898 (65%)
Prefer not to say	13 (.4%)
**Age**	**Mean = 37.15 (SD = 12.68)**
18–24	491 (17%)
25–34	946 (32%)
35–44	665 (23%)
45–54	491 (17%)
55–64	247 (8%)
65+	78 (3%)
**Education**	
No formal qualifications	20 (1%)
Secondary Education (GCSE/O-Levels)	358 (12%)
Post-Secondary Education (College, A-Levels, NVQ3 or below, or similar)	638 (22%)
Vocational Qualification (Diploma, Certificate, BTEC, NVQ 4 and above, or similar)	318 (11%)
Undergraduate Degree (BA, BSc etc.)	1068 (37%)
Post-graduate Degree (MA, MSc etc.)	448 (15%)
Doctorate (PhD, MD)	68 (2%)
**Household income**	
Less than £10,000	216 (7%)
£10,000 - £15,999	265 (9%)
£16,000 - £19,999	224 (8%)
£20,000 - £29,999	526 (18%)
£30,000 - £39,999	467 (16%)
£40,000 - £49,999	360 (12%)
£50,000 - £59,999	214 (7%)
£60,000 - £69,999	145 (5%)
£70,000 - £79,999	100 (3%)
£80,000 - £89,999	49 (2%)
£90,000 - £99,999	36 (1%)
£100,000 - £149,999	46 (2%)
More than £150,000	22 (1%)
Rather not say	247 (8%)

#### Procedure

The study received ethical approval from the Department of Security and Crime science at University College London. The study was hosted online at Qualtrics.com which was accessible via the recruitment platform. Participants were instructed to complete the survey via a desktop or laptop computer to allow the choice sets to display correctly across the screen. Participants were told that the study was about people’s decision making during the purchasing of internet connected products, what the study involved, that their data would be stored confidentially and that they had a right to withdraw at any time. Subsequently, they were asked to tick a box to indicate their consent to take part in the study. Participants were then randomly allocated to one of the seven labelling conditions and to two of the four IoT products. They were instructed to act as though they were thinking of purchasing the product and to make choices that they would in real life. They were provided with a brief explanation of the labelling condition they were allocated to but were not informed that the study was explicitly intended to assess the impact of it. They were also presented with a “cheap talk” script in which we explained the hypothetical bias to participants so as to reduce the probability that it would influence their choices [49]. Participants were presented with 20 choice sets in total (10 per product) and asked to choose between three product alternatives, or an opt out [50].

Upon completion of the choice sets, participants answered questions about their perceptions of the labelling scheme. First, using a seven-point rating scale (strongly agree to strongly disagree), they were asked to indicate whether they agreed or disagreed with the following statements: 1) that products which displayed the label used would protect them from online threats (such as hacking); 2) that the security label was appealing; 3) that the label was easy to understand; 4) that there was too much content to the label; 5) that they would use that type of label to help them when buying a product; and, 6) that the label would make it easy to compare products. Second, they were asked an open question about whether there was anything that they would change about the label. And, third, as we were interested in whether particular label designs might convey a false sense of security, we asked participants to indicate (on an eleven-point scale with 10% increments from 0–100%) the likelihood that a device carrying the label concerned would be vulnerable to hacking.

Finally, participants completed the SeBIS scale, a series of questions about themselves (demographics and whether they already owned IoT devices) and were debriefed about the study. In addition to the above, we included four attention checks to ensure that participants were adequately paying attention throughout the study. Two of these were presented after the instructions regarding the labelling content, and asked participants to select a correct explanation of what the label meant. The final two checks were presented in the final section of the study.

Before presenting the results, for the benefit of the reader, Table 3 provides a list of acronyms and terms used throughout the paper, along with a brief description of what they mean.

**Table 3:** Table of acronyms and terms.

Term	Definition
IoT	Internet of Things: Internet connected devices that can collect and share data over networks
WTP	Willingness to Pay: Maximum amount consumers are willing to pay for a product
DCE	Discrete Choice Experiment: method for estimating people’s preferences for particular attributes of a product experimentally
DCMS	UK Government Department for Digital, Culture, Media and Sport
SeBIS scale	Security Behaviour Intentions Scale used to measure participants existing security behaviour
SbD	Secure by Design (binary) seal of approval label
Graded label	Security is measured across a continuum but the result is simplified using a small number of “grades” (e.g. A to F)
Informational label	Communicates important information about a number of dimensions of security (e.g. for how long security updates will be provided)

## Results

### Effect of labels on stated preferences

To examine the influence of the labelling schemes on choice, the data were analysed using a mixed logit model. Similar to the conditional logit model commonly used in DCEs, the aim is to simultaneously estimate the influence of a range of factors (here, functionality, the presence and absence of a label, and price) on participant’s decision making. For both models it is assumed that a decision maker will select from a set of alternatives that for which they derive the most utility. That is, for a decision maker *n* who must make a choice *i* from *j* alternatives on *t* occasions, we assume that the utility *U*_*nit*_ > *U*_*njt*_ for all *i* ≠ *j*. For both models, utility is modelled as in Eq 1:

$$U_{nit}=V_{nit}+\varepsilon_{nit}$$ (1)

Where *V_nit_* is the utility associated with a systematic set of preferences (to be estimated), and *ε_nit_* is the utility gained from unobserved personal preferences and the idiosyncrasies of each participant.

While the conditional and mixed logit models are theoretically similar, they differ in (at least) three important ways. First, while the conditional logit model assumes that there is a single “fixed” effect for each variable, the mixed logit model assumes that there will be variability in the extent to which factors matter across participants and models this explicitly. Second, for the conditional logit to be valid, a number of assumptions—such as the independence of irrelevant alternatives (IIA, [51])—must be met. Such assumptions are commonly violated but the mixed logit relaxes these requirements, making it a more robust approach. And, third, unlike other logit models, the mixed logit cannot be solved analytically and must instead be estimated using maximum simulated likelihood (MSL) or other methods [52].

For the models tested, we estimate the effects of the presence or absence of a label, and variation in functionality and price. Price was modelled as a continuous linear function, while the label and functionality variables were modelled as binary variables (see Table 1).

WTP estimates were derived from the output of this model, and the approach taken to do this is discussed below. It is important to note that with the mixed logit model it is necessary to specify for which variables preferences might be expected to vary across participants. One approach is to assume that preferences will differ across participants for all variables. However, as part of our aim was to estimate WTP, it was necessary to estimate only a fixed effect for the price variable for that analysis, and hence for consistency we model price as a fixed effect in all analyses. We note, however, that doing so does not materially affect the results. A final point to note is that for MSL methods it is necessary to specify the number of Halton draws used for the simulation. Here, we use 500 Halton draws for all models as a sample of analyses suggested that the parameter estimates remained stable for 500 or more Halton draws. All analyses were conducted in STATA13SE.

In terms of presentation, we report the model (partial) coefficients—which estimate the relative importance of each factor—as odds ratios (ORs). These are multiplicative and show how much more likely a participant was to select a device in the presence of a label, or if a device had premium functionality, or for a one-unit change in price. With the exception of price, these effects are, of course, measured relative to a reference group. In the case of the security label, the reference group would be the same device without a label, while for premium functionality, the reference group would be the same device with standard functionality (see Table 1). In terms of interpretation, an OR of one indicates that a participant was no more likely to select a device with or without the feature of interest. ORs above one indicate that a participant would be that much more likely to select the device with the feature of interest, while those below one show how less likely a participant would be to select a device with a particular feature.

**Fig 3:**
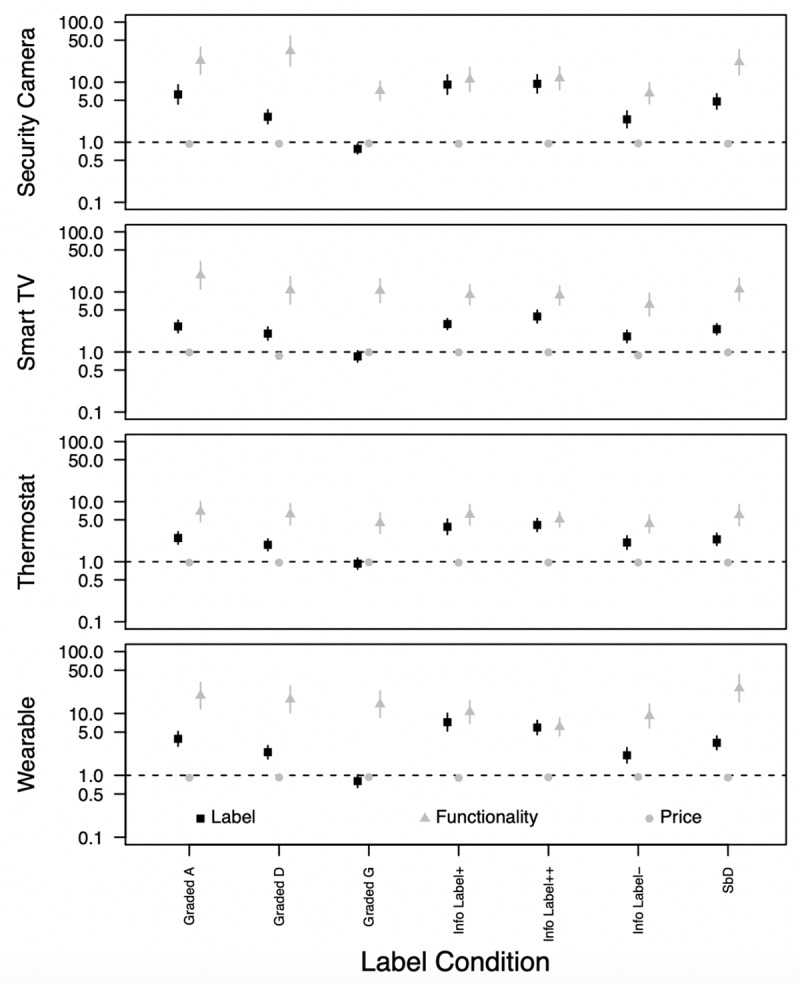
Odds ratios of the effects of labels, functionality and price on participant choice. NOTE: vertical bars show the 95% confidence intervals. Data are presented on a logarithmic scale for presentational purposes.

Analyses were conducted separately for each device and all label conditions. For parsimony, and to aid interpretation, we present the findings as a forest plot. Fig 3 shows the main effects of the variables discussed above. In each case, it is apparent that—all else equal—participants preferred devices that cost less. That is, the odds ratio for the price variable was always below one and always statistically significant (Mean OR = 0.95). However, as we will see below, participants were prepared to pay more for improved functionality and security.

Functionality clearly had a large influence on consumer choice. The unweighted mean OR of 11.8 (computed across all devices and all conditions), for example, suggests that relative to a device with basic functionality, participants were (on average) about 12 times more likely to select a device with a premium specification. The influence of functionality was significant for all devices but lower for thermostats than the other devices. The effect of functionality was also relatively stable across labelling conditions.

With respect to the effect of labels, this varied across conditions. With respect to the Graded labels, it is clear that participants were, on average, more likely to select a device with a graded A label (unweighted mean OR = 3.56), or one with a graded D label (unweighted mean OR = 2.22). However, they were less likely to select a device with a graded G label (unweighted mean OR = 0.84) than they were one without it—though this effect was only statistically significant for security cameras (*p*<0.01) and marginally so for wearables (*p*<0.10). In terms of the informational labels, participants were, on average, more likely to select a device that carried such a label than one that did not. Of the three labels, they were least likely to select a device with a short support period and that shared data with third parties (labelled *info label-*, unweighted mean OR = 2.09). Relative to the latter, they were, on average, just under two-and-a-half times as likely to select devices for which there was a long support period and data was not shared with third parties (labelled *info label+*, unweighted mean OR = 5.21), and were (on average) slightly more likely to select a device that additionally included a security icon (labelled *info label++*, unweighted mean OR = 5.46). The binary Secure by Design label also had a positive effect for all devices (unweighted mean OR = 3.09), but the effect was weaker than that for the information labels. On the basis of these results, the informational label for devices with the best security and privacy features (Info Label++) had the greatest effect on participants stated preferences. This was followed by the Info Label+, and then the Graded A and SbD labels.

Interestingly, while the effect of functionality was generally larger than that of the security labels, for the informational labels, the confidence intervals for these two variables generally overlapped, suggesting that the influence of these two factors were approximately equal. The exception was for Smart TVs, for which functionality appeared to have a consistently larger influence.

In addition to computing models to estimate the main effects of the variables we manipulated experimentally (price, label, and functionality), we examined the statistical interaction between our main variable of interest—the security label—and age, gender and self-reported security behaviour (measured using the mean score on the SeBIS scale). Across the 28 models (4 devices x 7 label conditions), almost all of the interaction terms were non-significant and there was no systematic pattern to the results (see S1–S4 Tables). As such, we discuss these analyses no further and estimate WTP using the model of main effects.

### Willingness to pay

It is well established that WTP can be estimated using results derived from a discrete choice modelling framework [53]. In this context, WTP for improvement in a certain attribute (e.g. functionality, security of IoT product) can be obtained as the ratio of the coefficient for that attribute and the overall cost coefficient. However, different approaches can be taken to estimate the standard error of the estimates. Here, we use the Delta method—implemented in STATA13SE—which is known [53] to produce precise estimates when the model is computed using a large sample, as is the case in this study.

**Fig 4:**
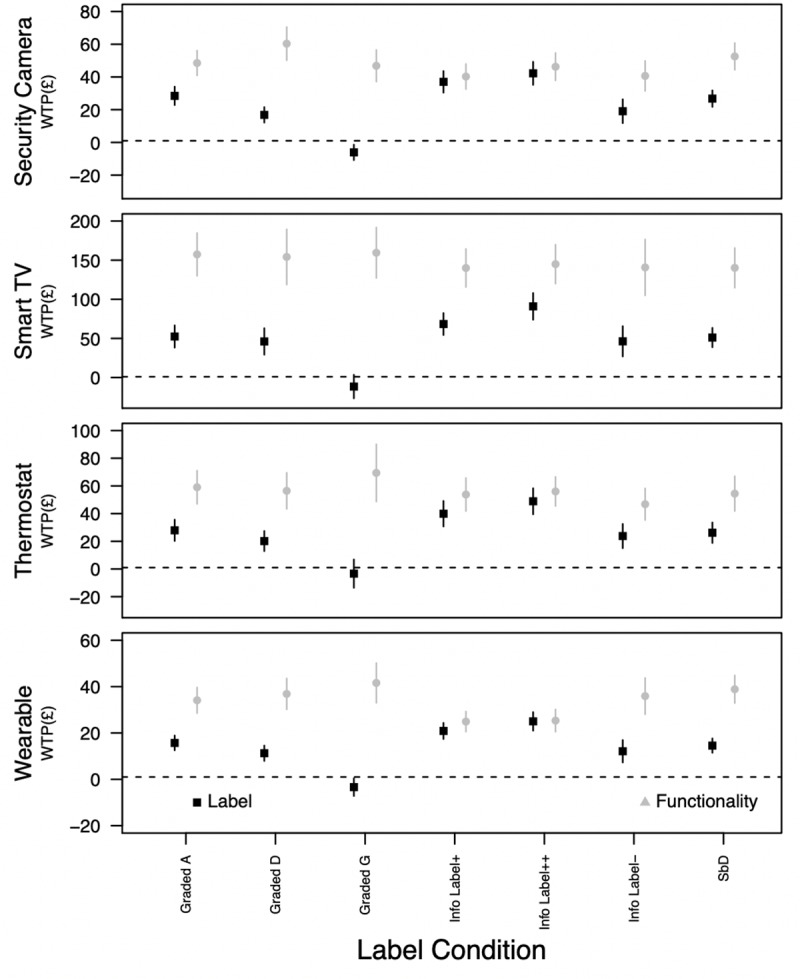
Participants willingness to pay (WTP) for different IoT labelling schemes and functionality. NOTE: vertical bars show the 95% confidence intervals.

Fig 4 shows participants’ WTP for the IoT devices tested for each labelling scheme and for premium functionality. As expected, respondents were willing to pay more for devices with higher specifications. Specifically, they were willing to pay, on average, an additional £48 (SD = 6.5), £148 (SD = 8.0), £34 (SD = 6.0) and £57 (SD = 6.3) for better functioning security cameras, Smart TVs, wearables and thermostats, respectively. This is between 29–40% of the average cost of the devices tested.

With the exception of the Graded G label, participants were also willing to pay more for devices that carried a security label. Relative to a device without a label, participants expected to pay between £3-£12 less for devices that carried the Graded G label. For two of the informational labels, and with the exception of Smart TVs, participants choices suggested that they would be willing to pay approximately the same additional cost for a device that carried a security label as they would for a premium device. In all other cases, participants were willing to pay between 27–63% (mean 40%) of what they were willing to pay for additional functionality.

#### Consumer preferences towards labelling options

After participants had completed the discrete choice experiment, they were asked a series of questions about the labelling option they were exposed to. For each item (with the exception of question 7), they were asked to rate the extent to which they agreed with the statement on a scale from 1 (strongly disagree) to 7 (strongly agree).

As shown in Table 4, perceptions of how the label would protect consumers was higher for the Graded A, followed by the SbD and Informational label with security assurances. For the Graded and Informational labels, the trends for the degree to which participants perceived that a device would protect them were in the expected directions (i.e. highest for Graded A and lowest for Graded G).

**Table 4:** Mean scores (and standard deviations) for participant’s preferences around labelling.

	Graded A label (n = 387)	Graded D Label (n = 389)	Graded G label (n = 426)	SbD (n = 437)	Info Label++ (n = 465)	Info Label+ (n = 428)	Info Label-(N = 386)
Protect me from online threats (such as hacking)	5.21 (1.36)	4.54 (1.40)	4.11 (1.81)	5.18 (1.32)	5.00 (1.49)	4.38 (1.59)	3.91 (1.69)
This label is appealing	5.55 (1.19)	5.02 (1.26)	4.96 (1.48)	4.78 (1.41)	5.46 (1.25)	5.28 (1.17)	5.19 (1.34)
This label is easy to understand	6.33 (.99)	5.96 (1.15)	6.04 (1.20)	5.61 (1.19)	6.33 (.83)	6.22 (.90)	6.24 (.86)
There is too much content on this label	2.10 (1.09)	2.22 (1.04)	2.18 (1.15)	2.75 (1.25)	2.40 (1.22)	2.31 (1.07)	2.35 (1.20)
I would use this type of label to help me when buying a product	5.60 (1.25)	5.21 (1.38)	5.43 (1.41)	5.09 (1.36)	5.90 (1.13)	5.74 (1.13)	5.65 (1.14)
This label would make it easy to compare products	5.88 (1.13)	5.50 (1.31)	5.65 (1.28)	5.01 (1.34)	5.92 (1.78)	5.78 (1.04)	5.77 (1.14)
On a scale of 0–100%, how likely do you think it is that a product that displays this label can be hacked	42.55 (28.33)	57.51 (24.40)	62.83 (26.98)	43.74 (26.40)	48.81 (27.68)	51.08 (25.96)	54.56 (25.87)

Most of the labels were considered fairly easy to understand, most were perceived to be at least somewhat appealing, and none were considered to contain too much information. Any differences observed were small. Participants generally thought that each of the labels would assist their purchasing decisions and make it easy to compare products, although the binary (SbD) label seemed to be perceived as slightly less useful in these respects.

In terms of the risk of hacking, participant’s expectations of risk were clearly correlated with the type of label. For example, participants estimated that a device carrying a Grade A label (43%) would be less susceptible to hacking than one that carried a Grade G label (63%). However, overall, participants tended not to think that any of the labels suggested a device was immune from hacking. In fact, for the labels perceived to convey the most security, participants thought there was more than a 40% chance that a device carrying that label could be hacked.

### Participants thoughts on label content

In addition to asking participants the closed questions discussed above, we invited them to provide qualitative comments about how they would improve the comprehension and clarity of the labelling options they were exposed to. To avoid attrition, participants were not required to answer these questions. Comments from those that did respond and that recurred are summarised below.

### Graded label

For the graded labelling conditions, 250 participants provided a response. Forty-eight percent of these concerned the need for further explanation of what the different grades meant, how the level of security was measured and what risks a device with this label would reduce. Three percent felt this information could be presented elsewhere, such as in a manual or on a website. Four percent of respondents wanted information about how the label was accredited (e.g. whether it was self-declared or independently verified). In terms of the design, 16% felt it was too similar to the energy label and that this may confuse consumers. Four percent thought the bands might be confusing as they get longer as the grading decreases and another 4% thought that less bands might be more helpful. Finally, 4% felt that the label would have to be mandatory to be effective.

### Secure by Design (SbD)

Seventy-two participants provided a response of which 29% wanted more explanation about the label and what risks it secures the consumer from. Seven percent felt that the label should refer to digital or cyber in order to avoid confusion with physical security. The remaining comments referred to the design of the label with 28% feeling that the design was slightly outdated and should be redesigned to give it a modern look. Like the graded label, 3% felt that the label should be mandatory.

### Information labels

Across the information label conditions, 111 participants provided a response. Twenty-one percent wanted further explanation of the label content, such as what information is shared with third parties and who these third parties are. Six percent felt that the security features needed better explanation. Other comments referred to the design with 14% wanting the use of more colour. Five percent felt the label could also provide details of the device’s specification and another 5% felt the warranty could be disclosed here. Finally, 5% thought that the label should be mandatory.

To get a general understanding of participants’ overall perceptions of the labelling options, we showed them generic versions of the three labels (Graded, Informational and SbD) and asked them “Of the three labels, which do you think would be most helpful to you to buy a secure product?”. We found that 46.5% preferred the informational label, 40.8% preferred the graded scheme, and 12.6% preferred the SbD label.

## Discussion

IoT devices vary considerably in the extent to which they provide security features to protect users from online threats. Furthermore, it is currently difficult (if possible) for consumers to differentiate devices that do and do not provide adequate levels of security at the point of purchase [11]. This creates a barrier to consumers adopting purchasing behaviours that would help increase their trust and protect them from cybercrime. One proposal to address this issue is for IoT devices to carry a security label to help consumers navigate the market and select devices that are most likely to protect them online. Using a discrete choice experiment, the aim of the present study was to estimate the potential impact of such labels on participant’s decision making, after controlling for the influence of device functionality and price. The use of such a study design also allowed us to estimate participant’s willingness to pay for devices that carry a security label without asking them this question directly, reducing the types of bias that plague other stated preference approaches to estimating WTP.

After controlling for the influence of price and functionality, with one (expected) exception, all of the security labels tested had a positive effect on participant’s choices. Neither age, gender or self-reported security behaviour seemed to affect whether participants were influenced by the security label or not. The most effective label tested was the informational label, for which two variants of this had the same influence on participant’s choices as the level of functionality of the product. In terms of these two versions of the label, the difference in the size of the effect each label had on participant’s choices was small. One reason for this may be that the difference between the two labels was too subtle. Specifically, while the most positive label indicated that the device had important security features, both labels had icons to indicate that security updates would be provided for a long period. It may be that participants believed the latter to indicate that devices carrying either label had good security features and as a result may not have perceived that the additional security icon conveyed much new information. Future research might explore this further and experiment with different icons.

This latter issue is also relevant to packaging design, since one of the places that such labels might appear is on the boxes that devices are shipped in. As boxes already carry a number of labels (e.g. CE and recycling labels), in order for a security label to influence consumer purchasing decisions, it will be important for the label to be designed and positioned in such a way that consumers can and will attend to it. Further research might usefully examine this issue, along with the other potential places such a label might appear. These might include websites, a removable sticker on the device itself, or shop pricing labels. If a label were implemented, it would also be important to ensure that sales staff understand what it conveys and can articulate this to consumers.

Considering participants willingness to pay, our estimates suggest that consumers are willing to pay more for IoT devices with premium functionality and those that carry a security label. Considering the amount that they would be willing to pay for increased security—the focus of this study—for the four labels that had the most positive effects on participant decision making (Graded A, Info Label+, Info Label++, and SbD), the average WTP estimates were £33.60, £65.71, £19.03 and £35.76 for security cameras, Smart TVs, Wearables, and Thermostats, respectively. For the label that had the largest effect on participant’s decision making—the informational label with an additional security icon—the same figures were £42.23, £90.95, £25.01 and £48.91, respectively. To put these figures into perspective, the mean price of these devices were £99.99, £350.99, £69.99 and £159.99 (see Table 1), respectively. As such, participants responses suggest that they would be willing to pay a non-trivial additional amount (both in terms of an absolute cost and a proportional increase) for devices that carried a security label. Moreover, for two of the informational labels, our estimates suggest that, with the exception of Smart TVs, participants would be willing to pay a very similar additional amount for a device that carried a security label as they would for one that had improved functionality. This suggests that cost is not necessarily a barrier to improving security in consumer IoT, at least in the proportions discussed above.

In terms of their responses to our open questions about the labels tested, participants reported that they would use them to inform purchasing decisions, and that they did not perceive them to be complicated. They did, however, report that they would like more explanation of the information the labels conveyed, with this perhaps being communicated in the device manuals or other materials. In terms of implementation, several participants noted that for a labelling scheme to work, it would need to be mandatory. This is an important issue. Even if a labelling scheme were not mandatory, consumers would need to be very much aware of it for it to have the potential to influence their behaviour (see [16]). Equally important was the finding that participants did not think that the security label implied that a device would be immune from hacking. If it did, this may have the unintended consequence of discouraging users from engaging in appropriate cyberhygiene.

Of course, our study is not without limitations. The most obvious is the fact that studies of this kind examine stated preferences by asking participants to say how they would act in a given scenario, and it is well known that stated preferences may differ to actual behaviours (e.g. [54]). To address this issue, researchers also study revealed preferences by analysing data on real purchases. While this approach enables the direct analysis of purchasing behaviour, it too is not without limitations. In the current context in particular, our aim was to estimate the potential effect of a labelling scheme that does not yet exist. For such research questions, there are no purchasing data and hence the analysis of revealed preferences is simply not possible. Should a label be implemented, then a study of revealed preferences would, of course, be the logical next step in research such as that reported here. While revealed preference studies offer greater ecological validity, it is, however, worth noting that they offer less control in terms of experimentation and implementation fidelity.

As discussed in the introduction, enhancing trust in the IoT is key to its adoption. Here, we explored labelling schemes as a means to indicate the trustworthiness of a product as it pertains to how the security requirements of the IoT product align with consumer need [4]. We did not, however, assess consumers’ perceived psychological trust in the type of consumer product, or factors that may influence this. That said, participants *were* given the option to choose between three products or to “opt out”—a task which represents a behavioural measure of consumer trust. Research on trust [55] indicates that there are three key factors that influence trust in IoT products: social-related factors, such as the extent to which the device aligns with their cultural values (e.g. a reserved culture may be less likely to adopt devices with video cameras than open cultures), and how a person’s social network perceives the device; security-related factors (such as risk and product security); and, product-related factors, such as usability. In our study, we explored the role of labelling to inform consumers security awareness of the product. Consequently, future research could usefully explore how social and product-related factors might mediate the impact of the labelling schemes on consumer choice and their willingness to pay. This would provide a greater understanding of the contextual factors that may impact on the efficacy of a security labelling scheme to enhance trust and aid consumer choice.

What the current study clearly suggests is that security labels have the potential to impact upon consumer choice and their willingness to pay for IoT devices. As such, the use of a security label to help consumers purchase more secure products appears to be a sensible policy option that should be tested further. In addition to impacting upon consumer behaviour directly, a labelling scheme also has the potential to incentivise industry to compete. For example, in the case of the energy label for white goods, due to market improvements in the energy efficiency of products since its introduction, this label has had to be updated to allow consumers to differentiate between goods with the highest levels of energy efficiency. In this way, the use of a label has the potential to act as a market lever that may encourage the manufacture of increasingly secure devices over time. At worst, it would explicitly draw industry and consumer attention to the issue.

## Supporting information

S1 Table Mixed Logit results for Smart TVs including interaction terms. (DOCX)

S2 Table Mixed Logit results for Thermostats including interaction terms. (DOCX)

S3 Table Mixed Logit results for Wearables including interaction terms. (DOCX)

S4 Table Mixed Logit results for security cameras including interaction terms. (DOCX)
