# Supplementary material for: The impact of IoT security labelling on consumer product choice and willingness to pay
Source: PLoS One. 2020 Jan 24;15(1):e0227800. doi: 10.1371/journal.pone.0227800 (PMC6980634; doi:10.1371/journal.pone.0227800)
Supplement: S1 Table — (DOCX) [file pone.0227800.s001.docx]

**Supporting Information**

|  | **Graded A** | | |  | **Graded D** | | |  | **Graded G** | | |  | **Info Label+** | | |  | **Info Label++** | | |  | **Info Label-** | | |  | **SbD** | | |
| --- | --- | --- | --- | --- | --- | --- | --- | --- | --- | --- | --- | --- | --- | --- | --- | --- | --- | --- | --- | --- | --- | --- | --- | --- | --- | --- | --- |
|  | B | s.e. | p |  | B | s.e. | p |  | B | s.e. | p |  | B | s.e. | p |  | B | s.e. | p |  | B | s.e. | p |  | B | s.e. | p |
| **Mean** |  |  |  |  |  |  |  |  |  |  |  |  |  |  |  |  |  |  |  |  |  |  |  |  |  |  |  |
| Price | -0.02 | 0.00 | 0.00 |  | -0.02 | 0.00 | 0.00 |  | -0.01 | 0.00 | 0.00 |  | -0.02 | 0.00 | 0.00 |  | -0.01 | 0.00 | 0.00 |  | -0.01 | 0.00 | 0.00 |  | -0.02 | 0.00 | 0.00 |
| Function | 2.97 | 0.27 | 0.00 |  | 2.38 | 0.27 | 0.00 |  | 2.35 | 0.23 | 0.00 |  | 2.21 | 0.19 | 0.00 |  | 2.16 | 0.19 | 0.00 |  | 1.79 | 0.23 | 0.00 |  | 2.39 | 0.22 | 0.00 |
| Label | 0.34 | 0.90 | 0.71 |  | 1.13 | 0.77 | 0.14 |  | -0.18 | 0.72 | 0.81 |  | -0.09 | 0.65 | 0.89 |  | -0.32 | 0.72 | 0.66 |  | 1.03 | 0.80 | 0.20 |  | 2.03 | 0.65 | 0.00 |
| MaleXLabel | 0.15 | 0.26 | 0.56 |  | -0.42 | 0.26 | 0.10 |  | 0.56 | 0.23 | 0.02 |  | -0.38 | 0.22 | 0.08 |  | -0.06 | 0.24 | 0.80 |  | 0.28 | 0.25 | 0.27 |  | -0.41 | 0.22 | 0.06 |
| AgeXLabel | 0.03 | 0.01 | 0.01 |  | 0.00 | 0.01 | 0.83 |  | 0.00 | 0.01 | 0.98 |  | 0.00 | 0.01 | 0.70 |  | 0.01 | 0.01 | 0.28 |  | 0.00 | 0.01 | 0.66 |  | 0.02 | 0.01 | 0.05 |
| SecXLabel | 0.15 | 0.24 | 0.54 |  | -0.08 | 0.22 | 0.72 |  | -0.05 | 0.20 | 0.82 |  | 0.37 | 0.19 | 0.05 |  | 0.48 | 0.21 | 0.02 |  | -0.14 | 0.22 | 0.50 |  | -0.28 | 0.18 | 0.11 |
|  |  |  |  |  |  |  |  |  |  |  |  |  |  |  |  |  |  |  |  |  |  |  |  |  |  |  |  |
| **SD** |  |  |  |  |  |  |  |  |  |  |  |  |  |  |  |  |  |  |  |  |  |  |  |  |  |  |  |
| Function | 2.91 | 0.25 | 0.00 |  | 2.82 | 0.28 | 0.00 |  | 2.59 | 0.24 | 0.00 |  | 2.35 | 0.19 | 0.00 |  | 2.27 | 0.18 | 0.00 |  | 2.26 | 0.23 | 0.00 |  | 2.68 | 0.22 | 0.00 |
| Label | -1.00 | 0.43 | 0.02 |  | -0.96 | 0.26 | 0.00 |  | -0.82 | 0.43 | 0.06 |  | -0.49 | 0.62 | 0.43 |  | -1.27 | 0.15 | 0.00 |  | -0.04 | 0.60 | 0.94 |  | 0.65 | 0.36 | 0.07 |
| Label_M | 0.67 | 0.55 | 0.22 |  | -0.05 | 0.37 | 0.90 |  | 0.04 | 0.39 | 0.92 |  | 0.00 | 0.37 | 1.00 |  | -0.06 | 0.48 | 0.90 |  | -0.37 | 0.90 | 0.68 |  | 0.22 | 0.57 | 0.70 |
| AgeXlabel | 0.03 | 0.02 | 0.12 |  | -0.04 | 0.02 | 0.04 |  | -0.01 | 0.03 | 0.71 |  | 0.01 | 0.03 | 0.79 |  | -0.02 | 0.03 | 0.61 |  | 0.00 | 0.04 | 0.98 |  | -0.01 | 0.03 | 0.78 |
| SecXLabel | 0.15 | 0.15 | 0.32 |  | 0.10 | 0.15 | 0.51 |  | 0.18 | 0.14 | 0.21 |  | 0.26 | 0.10 | 0.01 |  | -0.01 | 0.24 | 0.95 |  | 0.25 | 0.05 | 0.00 |  | 0.18 | 0.09 | 0.04 |
|  |  |  |  |  |  |  |  |  |  |  |  |  |  |  |  |  |  |  |  |  |  |  |  |  |  |  |  |
| Log-Likelihood | -1233.32 |  |  |  | -1244.32 |  |  |  | -1276.19 |  |  |  | -1529.54 |  |  |  | -1542.29 |  |  |  | -1011.84 |  |  |  | -1398.37 |  |  |
| N | 195 |  |  |  | 164 |  |  |  | 196 |  |  |  | 233 |  |  |  | 233 |  |  |  | 155 |  |  |  | 217.00 |  |  |

**Table S1** Mixed Logit results for Smart TVs including interaction terms (NOTE: the mean value is the mean (of the distribution of) raw beta coefficient estimated by the mixed logit model; SD is the standard deviation of the estimated model coefficients; SecXLabel models the interaction between self-reported security behaviour and the security the label)
