# Supplementary material for: The impact of IoT security labelling on consumer product choice and willingness to pay
Source: PLoS One. 2020 Jan 24;15(1):e0227800. doi: 10.1371/journal.pone.0227800 (PMC6980634; doi:10.1371/journal.pone.0227800)
Supplement: S2 Table — (DOCX) [file pone.0227800.s002.docx]

**Supporting Information**

|  | **Graded A** | | |  | **Graded D** | | |  | **Graded G** | | |  | **Info Label+** | | |  | **Info Label++** | | |  | **Info Label-** | | |  | **SbD** | | |
| --- | --- | --- | --- | --- | --- | --- | --- | --- | --- | --- | --- | --- | --- | --- | --- | --- | --- | --- | --- | --- | --- | --- | --- | --- | --- | --- | --- |
|  | B | s.e. | p |  | B | s.e. | p |  | B | s.e. | p |  | B | s.e. | p |  | B | s.e. | p |  | B | s.e. | p |  | B | s.e. | p |
| **Mean** |  |  |  |  |  |  |  |  |  |  |  |  |  |  |  |  |  |  |  |  |  |  |  |  |  |  |  |
| Price | -0.03 | 0.00 | 0.00 |  | -0.03 | 0.00 | 0.00 |  | -0.02 | 0.00 | 0.00 |  | -0.03 | 0.00 | 0.00 |  | -0.03 | 0.00 | 0.00 |  | -0.03 | 0.00 | 0.00 |  | -0.03 | 0.00 | 0.00 |
| Function | 1.95 | 0.20 | 0.00 |  | 1.83 | 0.21 | 0.00 |  | 1.47 | 0.20 | 0.00 |  | 1.79 | 0.20 | 0.00 |  | 1.63 | 0.15 | 0.00 |  | 1.45 | 0.18 | 0.00 |  | 1.80 | 0.21 | 0.00 |
| Label | -0.27 | 0.66 | 0.68 |  | 1.57 | 0.82 | 0.06 |  | -0.77 | 0.71 | 0.28 |  | 1.22 | 0.98 | 0.21 |  | 0.70 | 0.82 | 0.39 |  | 1.40 | 0.93 | 0.13 |  | 0.50 | 0.78 | 0.52 |
| MaleXLabel | 0.41 | 0.25 | 0.10 |  | -0.03 | 0.23 | 0.90 |  | -0.22 | 0.23 | 0.36 |  | 0.01 | 0.30 | 0.97 |  | -0.47 | 0.25 | 0.06 |  | 0.06 | 0.28 | 0.83 |  | -0.26 | 0.25 | 0.29 |
| AgeXLabel | 0.01 | 0.01 | 0.18 |  | 0.01 | 0.01 | 0.41 |  | 0.01 | 0.01 | 0.56 |  | 0.00 | 0.01 | 0.93 |  | 0.00 | 0.01 | 0.91 |  | 0.00 | 0.01 | 0.88 |  | 0.01 | 0.01 | 0.19 |
| SecXLabel | 0.29 | 0.19 | 0.13 |  | -0.25 | 0.22 | 0.26 |  | 0.21 | 0.20 | 0.29 |  | 0.04 | 0.28 | 0.89 |  | 0.24 | 0.23 | 0.29 |  | -0.18 | 0.25 | 0.47 |  | 0.13 | 0.22 | 0.57 |
|  |  |  |  |  |  |  |  |  |  |  |  |  |  |  |  |  |  |  |  |  |  |  |  |  |  |  |  |
| **SD** |  |  |  |  |  |  |  |  |  |  |  |  |  |  |  |  |  |  |  |  |  |  |  |  |  |  |  |
| Function | 2.11 | 0.19 | 0.00 |  | 2.29 | 0.21 | 0.00 |  | 2.29 | 0.22 | 0.00 |  | 2.20 | 0.20 | 0.00 |  | 1.71 | 0.15 | 0.00 |  | 1.79 | 0.18 | 0.00 |  | 2.47 | 0.22 | 0.00 |
| Label | 0.09 | 0.80 | 0.92 |  | 1.03 | 0.14 | 0.00 |  | 0.31 | 0.59 | 0.61 |  | -0.96 | 0.38 | 0.01 |  | 0.42 | 0.70 | 0.55 |  | 0.84 | 0.35 | 0.02 |  | -1.10 | 0.25 | 0.00 |
| Label_M | -0.27 | 0.41 | 0.52 |  | 0.05 | 0.37 | 0.88 |  | 0.61 | 0.46 | 0.18 |  | 0.06 | 0.64 | 0.93 |  | 0.02 | 0.43 | 0.96 |  | -0.51 | 0.58 | 0.38 |  | 0.00 | 0.43 | 1.00 |
| AgeXlabel | 0.00 | 0.02 | 0.97 |  | 0.00 | 0.02 | 0.93 |  | 0.02 | 0.03 | 0.58 |  | 0.04 | 0.03 | 0.13 |  | 0.04 | 0.03 | 0.19 |  | 0.07 | 0.03 | 0.01 |  | 0.02 | 0.08 | 0.79 |
| SecXLabel | -0.30 | 0.04 | 0.00 |  | -0.01 | 0.13 | 0.91 |  | 0.24 | 0.07 | 0.00 |  | 0.30 | 0.09 | 0.00 |  | 0.35 | 0.08 | 0.00 |  | 0.13 | 0.14 | 0.37 |  | -0.09 | 0.16 | 0.58 |
|  |  |  |  |  |  |  |  |  |  |  |  |  |  |  |  |  |  |  |  |  |  |  |  |  |  |  |  |
| Log-Likelihood | -1240.28 |  |  |  | -1212.59 |  |  |  | -1214.24 |  |  |  | -1224.69 |  |  |  | -1566.66 |  |  |  | -1142.14 |  |  |  | -1385.18 |  |  |
| N | 182 |  |  |  | 178 |  |  |  | 182 |  |  |  | 189 |  |  |  | 233 |  |  |  | 168 |  |  |  | 198 |  |  |

**Table S2** Mixed Logit results for Thermostats including interaction terms (NOTE: the mean value is the mean (of the distribution of) raw beta coefficient estimated by the mixed logit model; SD is the standard deviation of the estimated model coefficients; SecXLabel models the interaction between self-reported security behaviour and the security the label)
