# Supplementary material for: The impact of IoT security labelling on consumer product choice and willingness to pay
Source: PLoS One. 2020 Jan 24;15(1):e0227800. doi: 10.1371/journal.pone.0227800 (PMC6980634; doi:10.1371/journal.pone.0227800)
Supplement: S3 Table — (DOCX) [file pone.0227800.s003.docx]

**Supporting Information**

|  | **Graded A** | | |  | **Graded D** | | |  | **Graded G** | | |  | **Info Label+** | | |  | **Info Label++** | | |  | **Info Label-** | | |  | **SbD** | | |
| --- | --- | --- | --- | --- | --- | --- | --- | --- | --- | --- | --- | --- | --- | --- | --- | --- | --- | --- | --- | --- | --- | --- | --- | --- | --- | --- | --- |
|  | B | s.e. | p |  | B | s.e. | p |  | B | s.e. | p |  | B | s.e. | p |  | B | s.e. | p |  | B | s.e. | p |  | B | s.e. | p |
| **Mean** |  |  |  |  |  |  |  |  |  |  |  |  |  |  |  |  |  |  |  |  |  |  |  |  |  |  |  |
| Price | -0.09 | 0.00 | 0.00 |  | -0.08 | 0.00 | 0.00 |  | -0.06 | 0.00 | 0.00 |  | -0.09 | 0.00 | 0.00 |  | -0.07 | 0.00 | 0.00 |  | -0.06 | 0.00 | 0.00 |  | -0.08 | 0.00 | 0.00 |
| Function | 2.98 | 0.26 | 0.00 |  | 2.85 | 0.26 | 0.00 |  | 2.67 | 0.26 | 0.00 |  | 2.35 | 0.22 | 0.00 |  | 1.81 | 0.17 | 0.00 |  | 2.25 | 0.23 | 0.00 |  | 3.25 | 0.26 | 0.00 |
| Label | 0.57 | 0.84 | 0.50 |  | -0.19 | 0.73 | 0.79 |  | -0.67 | 0.78 | 0.39 |  | -1.30 | 0.90 | 0.15 |  | 1.28 | 0.84 | 0.13 |  | 2.40 | 0.96 | 0.01 |  | 0.66 | 0.75 | 0.38 |
| MaleXLabel | 0.30 | 0.29 | 0.30 |  | 0.46 | 0.31 | 0.13 |  | 0.37 | 0.28 | 0.18 |  | -0.06 | 0.32 | 0.86 |  | -0.23 | 0.25 | 0.36 |  | 0.06 | 0.31 | 0.86 |  | -0.03 | 0.27 | 0.90 |
| AgeXLabel | 0.02 | 0.01 | 0.10 |  | 0.01 | 0.01 | 0.24 |  | -0.01 | 0.01 | 0.56 |  | -0.01 | 0.01 | 0.46 |  | 0.01 | 0.01 | 0.51 |  | -0.01 | 0.01 | 0.46 |  | 0.03 | 0.01 | 0.01 |
| SecXLabel | 0.18 | 0.24 | 0.43 |  | 0.25 | 0.21 | 0.23 |  | 0.09 | 0.22 | 0.67 |  | 0.93 | 0.26 | 0.00 |  | 0.16 | 0.24 | 0.49 |  | -0.46 | 0.26 | 0.08 |  | 0.16 | 0.21 | 0.44 |
|  |  |  |  |  |  |  |  |  |  |  |  |  |  |  |  |  |  |  |  |  |  |  |  |  |  |  |  |
| **SD** |  |  |  |  |  |  |  |  |  |  |  |  |  |  |  |  |  |  |  |  |  |  |  |  |  |  |  |
| Function | 2.71 | 0.24 | 0.00 |  | 2.87 | 0.26 | 0.00 |  | 2.63 | 0.25 | 0.00 |  | 2.46 | 0.22 | 0.00 |  | 2.13 | 0.17 | 0.00 |  | 2.43 | 0.24 | 0.00 |  | 3.01 | 0.25 | 0.00 |
| Label | 0.39 | 0.81 | 0.63 |  | -0.92 | 0.22 | 0.00 |  | 0.59 | 0.39 | 0.14 |  | -0.79 | 0.51 | 0.12 |  | 1.21 | 0.27 | 0.00 |  | 0.98 | 0.37 | 0.01 |  | -1.39 | 0.16 | 0.00 |
| Label_M | -0.36 | 0.80 | 0.66 |  | 1.58 | 0.35 | 0.00 |  | -1.13 | 0.30 | 0.00 |  | 0.39 | 1.11 | 0.73 |  | -0.23 | 0.54 | 0.67 |  | -0.05 | 0.91 | 0.95 |  | -0.31 | 0.79 | 0.69 |
| AgeXlabel | 0.04 | 0.03 | 0.10 |  | 0.02 | 0.03 | 0.50 |  | 0.06 | 0.02 | 0.00 |  | 0.03 | 0.04 | 0.38 |  | -0.04 | 0.02 | 0.12 |  | -0.03 | 0.05 | 0.58 |  | -0.01 | 0.04 | 0.88 |
| SecXLabel | 0.32 | 0.09 | 0.00 |  | -0.05 | 0.14 | 0.70 |  | -0.12 | 0.13 | 0.36 |  | 0.41 | 0.10 | 0.00 |  | 0.19 | 0.12 | 0.12 |  | 0.27 | 0.11 | 0.01 |  | -0.04 | 0.15 | 0.79 |
|  |  |  |  |  |  |  |  |  |  |  |  |  |  |  |  |  |  |  |  |  |  |  |  |  |  |  |  |
| Log-Likelihood | -1269.90 |  |  |  | -1347.07 |  |  |  | -1161.00 |  |  |  | -1314.70 |  |  |  | -1582.37 |  |  |  | -1179.40 |  |  |  | -1528.48 |  |  |
| N | 200.00 |  |  |  | 210.00 |  |  |  | 181.00 |  |  |  | 214.00 |  |  |  | 243.00 |  |  |  | 181 |  |  |  | 235.00 |  |  |

**Table S3** Mixed Logit results for Wearables including interaction terms (NOTE: the mean value is the mean (of the distribution of) raw beta coefficient estimated by the mixed logit model; SD is the standard deviation of the estimated model coefficients; SecXLabel models the interaction between self-reported security behaviour and the security the label)
