# Supplementary material for: The impact of IoT security labelling on consumer product choice and willingness to pay
Source: PLoS One. 2020 Jan 24;15(1):e0227800. doi: 10.1371/journal.pone.0227800 (PMC6980634; doi:10.1371/journal.pone.0227800)
Supplement: S4 Table — (DOCX) [file pone.0227800.s004.docx]

**Supporting Information**

|  | **Graded A** | | |  | **Graded D** | | |  | **Graded G** | | |  | **Info Label+** | | |  | **Info Label++** | | |  | **Info Label-** | | |  | **SbD** | | |
| --- | --- | --- | --- | --- | --- | --- | --- | --- | --- | --- | --- | --- | --- | --- | --- | --- | --- | --- | --- | --- | --- | --- | --- | --- | --- | --- | --- |
|  | B | s.e. | p |  | B | s.e. | p |  | B | s.e. | p |  | B | s.e. | p |  | B | s.e. | p |  | B | s.e. | p |  | B | s.e. | p |
| **Mean** |  |  |  |  |  |  |  |  |  |  |  |  |  |  |  |  |  |  |  |  |  |  |  |  |  |  |  |
| Price | -0.06 | 0.00 | 0.00 |  | -0.06 | 0.00 | 0.00 |  | -0.04 | 0.00 | 0.00 |  | -0.06 | 0.00 | 0.00 |  | -0.05 | 0.00 | 0.00 |  | -0.05 | 0.00 | 0.00 |  | -0.06 | 0.00 | 0.00 |
| Function | 3.13 | 0.27 | 0.00 |  | 3.55 | 0.30 | 0.00 |  | 1.97 | 0.19 | 0.00 |  | 2.40 | 0.24 | 0.00 |  | 2.46 | 0.23 | 0.00 |  | 1.91 | 0.21 | 0.00 |  | 3.06 | 0.25 | 0.00 |
| Label | 0.63 | 1.13 | 0.58 |  | -0.27 | 0.81 | 0.74 |  | -1.06 | 0.67 | 0.11 |  | 1.89 | 1.18 | 0.11 |  | 0.82 | 1.03 | 0.43 |  | 0.87 | 1.08 | 0.42 |  | 1.48 | 0.92 | 0.11 |
| MaleXLabel | 0.13 | 0.36 | 0.72 |  | 0.27 | 0.31 | 0.39 |  | -0.16 | 0.22 | 0.47 |  | 0.23 | 0.37 | 0.53 |  | -0.19 | 0.36 | 0.59 |  | -0.01 | 0.37 | 0.97 |  | 0.33 | 0.31 | 0.29 |
| AgeXLabel | 0.00 | 0.02 | 0.81 |  | 0.00 | 0.01 | 0.98 |  | -0.01 | 0.01 | 0.29 |  | 0.00 | 0.01 | 0.73 |  | -0.03 | 0.01 | 0.04 |  | -0.03 | 0.01 | 0.05 |  | 0.01 | 0.01 | 0.36 |
| SecXLabel | 0.33 | 0.32 | 0.31 |  | 0.31 | 0.22 | 0.16 |  | 0.24 | 0.19 | 0.21 |  | 0.07 | 0.33 | 0.82 |  | 0.41 | 0.29 | 0.15 |  | -0.01 | 0.29 | 0.99 |  | -0.01 | 0.26 | 0.96 |
|  |  |  |  |  |  |  |  |  |  |  |  |  |  |  |  |  |  |  |  |  |  |  |  |  |  |  |  |
| **SD** |  |  |  |  |  |  |  |  |  |  |  |  |  |  |  |  |  |  |  |  |  |  |  |  |  |  |  |
| Function | 2.56 | 0.25 | 0.00 |  | 3.06 | 0.29 | 0.00 |  | 2.15 | 0.19 | 0.00 |  | 2.66 | 0.23 | 0.00 |  | 2.40 | 0.22 | 0.00 |  | 2.11 | 0.21 | 0.00 |  | 2.71 | 0.25 | 0.00 |
| Label | 1.11 | 0.40 | 0.01 |  | -1.01 | 0.39 | 0.01 |  | 0.20 | 0.51 | 0.70 |  | 1.94 | 0.24 | 0.00 |  | 1.59 | 0.24 | 0.00 |  | 1.13 | 0.36 | 0.00 |  | -1.09 | 0.39 | 0.01 |
| Label_M | -1.01 | 0.52 | 0.05 |  | 1.58 | 0.38 | 0.00 |  | 0.71 | 0.27 | 0.01 |  | -0.37 | 0.74 | 0.62 |  | 1.26 | 0.53 | 0.02 |  | -1.33 | 0.57 | 0.02 |  | 0.86 | 0.47 | 0.07 |
| AgeXlabel | 0.01 | 0.05 | 0.84 |  | -0.01 | 0.03 | 0.69 |  | 0.01 | 0.02 | 0.84 |  | 0.02 | 0.04 | 0.60 |  | 0.00 | 0.02 | 0.95 |  | -0.01 | 0.04 | 0.82 |  | -0.03 | 0.04 | 0.45 |
| SecXLabel | 0.35 | 0.12 | 0.01 |  | 0.07 | 0.37 | 0.85 |  | 0.23 | 0.05 | 0.00 |  | 0.18 | 0.13 | 0.15 |  | 0.08 | 0.17 | 0.63 |  | -0.26 | 0.14 | 0.05 |  | -0.24 | 0.14 | 0.08 |
|  |  |  |  |  |  |  |  |  |  |  |  |  |  |  |  |  |  |  |  |  |  |  |  |  |  |  |  |
| Log-Likelihood | -1116.75 |  |  |  | -1201.53 |  |  |  | -1402.61 |  |  |  | -1209.43 |  |  |  | -1200.47 |  |  |  | -1120.77 |  |  |  | -1341.40 |  |  |
| N | 175 |  |  |  | 193 |  |  |  | 201 |  |  |  | 197 |  |  |  | 196 |  |  |  | 166 |  |  |  | 206 |  |  |

**Table S4** Mixed Logit results for Security Cameras including interaction terms (NOTE: the mean value is the mean (of the distribution of) raw beta coefficient estimated by the mixed logit model; SD is the standard deviation of the estimated model coefficients; SecXLabel models the interaction between self-reported security behaviour and the security the label)
